# Results from a living systematic review of the prevalence of mood and anxiety disorders and factors associated with symptoms in systemic sclerosis

**DOI:** 10.1038/s41598-023-31919-8

**Published:** 2023-03-30

**Authors:** Elsa-Lynn Nassar, Dalal A. Abdulkareem, Brett D. Thombs

**Affiliations:** 1 Lady Davis Institute for Medical Research, Jewish General Hospital, 3755 Cote Ste Catherine Road, Pavilion H4.83, Montreal, QC H3T 1E2 Canada; 2 Department of Psychiatry, McGill University, Montreal, QC Canada; 3 Department of Epidemiology, Biostatistics, and Occupational Health, McGill University, Montreal, QC Canada; 4 Department of Psychology, McGill University, Montreal, QC Canada; 5 Department of Medicine, McGill University, Montreal, QC Canada; 6 Biomedical Ethics Unit, McGill University, Montreal, QC Canada

**Keywords:** Psychology, Diseases, Rheumatology, Risk factors, Signs and symptoms

## Abstract

We aimed to synthesize evidence on (1) the prevalence of mood and anxiety disorders and (2) factors associated with symptoms in systemic sclerosis (SSc). We searched MEDLINE, CINAHL, EMBASE, Cochrane CENTRAL, and PsycINFO via an ongoing living systematic review with automated monthly searches. We identified 6 eligible studies through March 1, 2023. Based on 3 studies (N = 93 to 345), current or 30-day major depressive disorder prevalence was 4% (95% confidence interval [CI] 2%, 6%) in a sample of Canadian outpatients (N = 345), 18% (95% CI 12%, 27%) in a study of Indian outpatients (N = 93), 10% (95% CI 4%, 21%) for French patient conference attendees (N = 51), and 29% (95% CI 18%, 42%) for French inpatients (N = 49). Current or 30-day prevalence of any anxiety disorder was 49% (95% CI 36%, 62%) for French conference attendees and 51% (95% CI 38%, 64%) for French inpatients; current or 30-day prevalence of generalized anxiety disorder was 3% for Indian outpatients (95% CI 1%, 9%; N = 93). In 3 studies (N = 114 to 376) that examined factors associated with depressive symptoms, higher education and being married or living as married were associated with lower symptoms and pulmonary involvement, breathing problems, and tender joint counts with higher symptoms; age and disease severity markers were not associated. Only 1 study (N = 114) assessed factors associated with anxiety symptoms and found no statistically significant associations. Limitations included heterogeneous populations and assessment methods, small samples, and substantial risk of bias concerns. Mood and anxiety disorder prevalence appear high in SSc, but estimates vary, and existing studies have important limitations. Future research should assess mood and anxiety prevalence and factors associated with symptoms using large representative samples and validated classification and assessment methods.

Review registration: PROSPERO (CRD 42021251339).

## Introduction

Systemic sclerosis (SSc; scleroderma) is a rare chronic, autoimmune rheumatic disease characterized by abnormal fibrotic processes and excessive collagen production, which manifests in skin thickening and fibrosis of internal organs, including the heart, lungs, and gastrointestinal tract^1,2^. SSc disease presentation is extremely heterogeneous, and its course is unpredictable^3,4^. Common symptoms include hand function and mobility limitations, pain, fatigue, gastrointestinal symptoms, pruritus, sleep problems, and mental health concerns, including body image distress from disfigurement (e.g., skin tightening, pigment changes, hand contractures, telangiectasias)^5^. People with SSc experience substantially lower health-related quality of life compared to the general population^6^ and people with other rheumatic diseases^7^. People with SSc may be at risk for depression and anxiety due to the unpredictable and progressive course of the disease^1,2^, high levels of chronic pain^8^, fatigue^9^, body-image distress^5,10^, overall disability, increased risk of mortality, and limited treatment options^1,2,9^.

No systematic reviews have examined prevalence of anxiety disorders or factors associated with anxiety symptoms in SSc. One systematic review, which included studies published up to 2006, examined depression prevalence and associated factors^11^. The review did not identify any studies that assessed prevalence of major depressive disorder (MDD) or other mood disorders established with validated diagnostic interview methods. Instead, it included studies that reported “prevalence” based on the proportion of individuals scoring above a cut-off score on a depression screening tool. However, since that review was conducted, it has become increasingly clear that using self-report questionnaires to generate “prevalence” estimates produces results that are highly exaggerated compared to validated methods based on diagnostic interviews^12–16^. Although the extent to which self-report questionnaires overestimate prevalence depends on the questionnaire and cut-off score used^12–16^, a series of 3 individual participant meta-analyses that included between 6,005 and 9,242 participants each found that estimated prevalence using standard cut-offs on the Hospital Anxiety and Depression Scale—Depression subscale, Patient Health Questionnaire-9, and Edinburgh Postnatal Depression Scale ranged from 25 to 28%, compared with 9 to– 12% based on validated diagnostic interview methods^14–16^. An additional limitation is that the authors could not draw conclusions about associated factors due to methodological limitations of included studies^11^.

Living systematic reviews are systematic reviews that are updated regularly to incorporate evidence as it becomes available^17,18^. They ensure timely access to evidence and reduce costs and delays from having to re-launch the review process from scratch when evidence becomes out of date^17,18^. Given the potentially high prevalence of depression and anxiety in people with SSc and the importance of understanding factors associated with symptoms for optimal health service delivery and management of SSc, we are conducting a living systematic review to assess (1) prevalence of mood and anxiety disorders and (2) factors associated with mood and anxiety symptoms in SSc. Our living systematic review approach is driven by several factors, including ongoing uncertainty in the evidence base, the need for timely access to evidence, and the likelihood of new evidence emerging that would inform clinical practice decisions^18^. The present report is the first evidence report from this living systematic review.

## Methods

Our living systematic review was registered in the PROSPERO prospective register of systematic reviews (CRD 42021251339), and a study protocol was developed and posted on the Open Science Framework prior to initiation (https://osf.io/fmtxp/). Results are reported following the Preferred Reporting Items for Systematic Reviews and Meta-analyses (PRISMA) statement^19^.

### Study eligibility

#### Prevalence of mood and anxiety disorders

Eligible studies are primary studies in any language that assessed the prevalence of mood or anxiety disorders among people with SSc. Studies are eligible if mood or anxiety disorder status were ascertained using a validated semi-structured or fully structured diagnostic interview method and Diagnostic and Statistical Manual (DSM) or International Classification of Diseases (ICD) criteria. Studies that reported “prevalence” based on other methods not valid for this purpose, such as unstructured diagnoses, self-report questionnaires, rating scales, or medical records, are excluded. Studies that included both participants with SSc and other conditions are included only if outcomes were reported separately for those with SSc or if participants with SSc comprised at least 80% of the study sample. Studies that included < 50 participants with SSc are not included due to their limited utility for attempting to estimate prevalence. Any studies that reported primary data, including conference abstracts, are eligible. Case studies, editorials, systematic reviews, and meta-analyses are excluded.

#### Factors associated with mood and anxiety symptoms

Eligible studies are primary studies published in any language that examined factors associated with mood or anxiety disorders or symptoms among at least 100 participants with SSc. Studies that included < 100 participants with SSc are not included since multivariate assessment of factors requires larger sample sizes to be useful. To be eligible, studies must have classified participants’ mood or anxiety disorder status using validated semi-structured or fully structured diagnostic interview methods and DSM or ICD criteria or assessed symptoms based on a validated self-report questionnaire. Studies that included both participants with SSc and other conditions are included only if outcomes were reported separately for those with SSc or if participants with SSc comprised at least 80% of the study sample. Studies must have conducted multivariate assessments of factors. If factors in a multivariate model included other concurrently measured mental health variables or other self-reported outcomes for which directionality with mental health symptoms was unclear (e.g., pain, fatigue, self-efficacy), the study is excluded. This is because, like depressive or anxiety symptoms, these variables are often outcomes of SSc and would be expected to have bidirectional causal associations with depressive and anxiety symptoms. When there is reverse causation in models, meaning that outcome variables might be causally linked to predictor variables, (1) all model coefficients might be biased, which could mask potentially important associations between disease variables and depressive and anxiety symptoms; (2) goodness-of-fit estimates (R^2^) are likely to be spuriously inflated; and (3) there is no way to determine the relative causal influence between the variables for which reverse causation is likely^20^. If a study used depressive or anxiety symptom levels as an eligibility criterion (e.g., analysis among people with high levels of depressive symptoms), it is excluded. Any studies that reported eligible primary data, including conference abstracts, are eligible. Case studies or reports, letters to the editor, systematic reviews, and meta-analyses are excluded.

### Search strategy

We have searched MEDLINE, EMBASE, Cochrane CENTRAL, CINAHL, and PsycINFO databases for relevant articles, using a strategy designed and built by an experienced health sciences librarian (see Online Appendix S1 for search terms). For depression, we first reviewed articles included in the previous systematic review, and we have searched for additional articles published since November 2, 2006, the end date of the previous systematic review search^11^. For anxiety, we have searched for articles on anxiety published since the inception date of each database. In addition to database searches, we review references from other relevant reviews and query authors of included studies about unpublished eligible studies. After the initial search, we set automated searches for monthly updates to facilitate continual review and update. We plan to review our search periodically to identify any terminology changes that should be incorporated. The last search for studies included in the present report was conducted on March 1, 2023, and we plan to incorporate evidence as new studies are identified.

### Selection of eligible studies

Search results are uploaded into the systematic review software DistillerSR (Evidence Partners, Ottawa, Canada), where duplicate references are identified and removed. Two investigators independently review studies for eligibility. If either reviewer deems a study potentially eligible based on title and abstract review, full‐text review is conducted, also independently by 2 reviewers. Discrepancies at the full-text level are resolved through consensus, with a third investigator consulted as necessary. To ensure the accurate identification of eligible studies, a coding guide with inclusion and exclusion criteria was developed and pretested (see Online Appendix S2).

### Data extraction

For each included study, 1 reviewer extracts the data using a pre-specified standardized form, and a second reviewer validates the extracted data using the DistillerSR Quality Control function (see Online Appendix S3). Any discrepancies are resolved by consensus between the 2 reviewers, involving a third reviewer if necessary. For each included study, we extract the (1) publication characteristics (i.e., first author last name, year of publication, journal, and publication year); (2) participant demographics (i.e., age, sex, recruitment method, sample size, number of included participants, disease duration); (3) outcomes of interest (i.e., prevalence of depression and anxiety or factors associated with depression and anxiety symptoms or disorders); and (4) risk of bias and adequacy of study methods and reporting. We calculate 95% confidence intervals (CIs) around prevalence estimates via the Agresti and Coull method^21^. Risk of bias and adequacy of study methods and reporting is assessed using an adapted version of the Joanna Briggs Institute Checklist for Prevalence Studies (see Online Appendices S4 and S5)^22^.

### Data analysis

Meta-analyses were not conducted for this report due to the small number of included studies and the high degree of heterogeneity in participant characteristics and methods. Study characteristics and outcomes were instead described qualitatively. If enough new evidence is identified of sufficiently adequate quality and low heterogeneity to synthesize quantitatively, we will conduct a random-effects meta-analysis of proportions to determine the pooled prevalence. To do this, we will use the metaprop command within R’s meta-package, which uses an inverse-variance meta-analysis method and logit transformation. To assess heterogeneity, we will calculate I^2^.

## Results

### Search results

**Figure 1:**
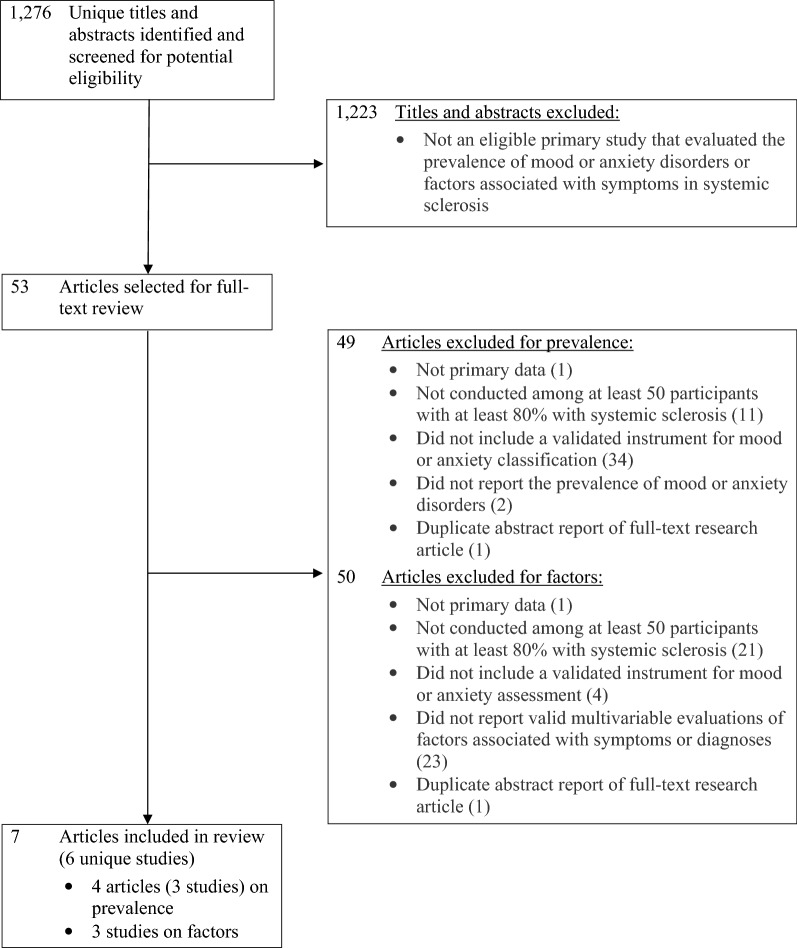
Flow diagram of selection of eligible studies.

The database search yielded 1276 unique titles and abstracts up to the March 1, 2023 search. Of these, 1223 were excluded after title and abstract review and 46 after full-text review, leaving 6 eligible primary studies, which were reported in 7 publications^23–29^ (Fig. 1). Of these, 2 studies^23,26^ assessed the prevalence of both depressive and anxiety disorders, 1^24,25^ assessed the prevalence of depressive disorders only, 1^27^ assessed factors associated with both depressive and anxiety symptoms, and 2^28,29^ assessed factors associated with depressive symptoms. No eligible studies were identified based on hand searches or backward searches of reference lists.

### Characteristics of included studies

All included studies required participants to meet Leroy and Medsger, 1980 American College of Rheumatology, or 2013 American College of Rheumatology / European League Against Rheumatism criteria for SSc classification. All included studies were cross-sectional except one with 2 assessments 1 month apart^24,25^. Characteristics of included studies are shown in Table 1.

**Table 1:** Included study characteristics.

First author (year)	Country	Dates of data collection	N	Setting and eligibility	Age in years: mean (SD)^a^	% Female	% diffuse subtype	Disease duration in years: mean (SD)^a^	Results included for prevalence	Results included for factors
Prevalence of mood or anxiety disorders										
Baubet (2011)^23^	France	05/2002–05/2004	100	49 adult inpatients and 51 attendees at a patient association meeting	Median = 53 (IQR 44–60)	86%	Not reported	Unspecified index event: median = 6 (IQR 2–10)	Depression Anxiety	–
Jewett/Thombs (2014/2015)^24,25^	Canada	04/2009–05/2012	345	Outpatients from multiple clinics	58 (12)	88%	24%	Diagnosis: 7 (8) Non-Raynaud’s symptom onset: 10 (10)	Depression	–
Jha (2022)^26^	India	08/2013–08/2017	93	Outpatients from a single tertiary care centre	42 (11)	86%	12%	Unspecified index event: 6 (range 1–22)	Depression Anxiety	–
Factors associated with mood or anxiety symptoms										
Faezi (2017)^27^	Iran	01/2013–01/2016	114	Adults aged 18–65 years with no documented history of major depressive or anxiety disorders prior to disease onset	39 (11)	89%	55%	Unspecified index event: 8 (2)	–	Depression Anxiety
Kwakkenbos (2012)^28^	Netherlands	06/2008–02/2010	215	Outpatients from 2 clinics	56 (12)	68%	25%	Non-Raynaud's symptom onset: 9 (8)	–	Depression
Thombs (2008)^29^	Canada	09/2004–10/2006	376	Outpatients from multiple clinics	55 (13)	87%	48%	Diagnosis: 9 (8) Non-Raynaud's symptom onset: 11 (9)	–	Depression

*IQR* inter-quartile range, *SD* standard deviation.

^a^Mean and standard deviation reported unless not available.

Three studies, published between 2011 and 2022, examined the prevalence of mood or anxiety disorders^23–26^. Of these, 1 study reported data from both inpatients recruited from hospitals in France and patients who attended a patient organization meeting^23^, 1 from outpatients recruited from multiple clinics in Canada^24,25^, and 1 from outpatients recruited from a tertiary care center in India^26^. Sample size was 345 in the Canadian study^24,25^ and between 49 and 93 in the 2 French samples^23^ and the sample from India^26^. Mean age ranged from 42 to 58 years, and the percentage of female participants in each study ranged from 86 to 88%. Mean disease duration ranged from 6 to 10 years. The proportion of participants with diffuse SSc was 24%^24,25^ in the Canadian study, 12% in the study from India^26^, and not reported in the French study^23^.

Three studies, published between 2008 and 2017, examined factors associated with depressive or anxiety symptoms^27–29^. Two studies reported data from outpatients in the Netherlands^28^ and Canada^29^, and 1 study on Iranian patients did not report setting^27^. The studies ranged in size from 114 to 376 participants. Mean age ranged from 39 to 56 years, and the percentage of female participants from 68 to 89%. Mean disease duration ranged from 8 to 11 years. The proportion of participants with diffuse SSc ranged from 25 to 55%.

### Risk of bias and adequacy of study methods and reporting

Ratings of adequacy of methods and reporting are shown in Online Appendix S6 for prevalence studies and Online Appendix S7 for studies on factors associated with symptoms.

Among the 3 prevalence studies^23–26^, in the French study, separate ratings were applied for patient conference attendee and inpatient samples^23^. Of the 4 samples, all 4 were rated “Yes” for appropriate statistical analysis; 3 of 4 for standard and reliable measurement; 1 of 4 for appropriate sampling frame, adequate sample size, detailed description of study subjects and setting, and adequate follow-up response rate and management; and none for recruitment method, adequate response rate and coverage, and methods used for the identification of mood or anxiety disorders.

For the 3 studies of factors associated with symptoms, all 3 were rated “Yes” for adequate coverage of potential predictors, valid methods used for the identification of symptom levels, and appropriate data presented for all variables; 2 of 3 for adequate sample size; 1 of 3 for appropriate participant recruitment, adequate response rate and management, and standard and reliable measurement; and none for pre-specification of regression model variables and appropriate sampling frame.

### Prevalence of mood disorders

Prevalence of mood and anxiety disorders is reported in Table 2. For one study from France, results are reported separately for inpatients recruited from hospitals and attendees at a patient association meeting if they were reported separately in the study publication but combined if not^23^. Current or 30-day MDD prevalence was 10% (95% CI 4%, 21%) for French patient conference attendees^23^ and 29% (95% CI 18%, 42%) for French inpatients^23^ assessed with the Mini International Neuropsychiatric Interview (MINI), 4% (95% CI 2%, 6%) for Canadian outpatients^24,25^ assessed with the Composite International Diagnostic Interview (CIDI), and 18% (95% CI 12%, 27%) for Indian outpatients assessed with the Revised Clinical Interview Schedule (CIS-R)^26^. Lifetime MDD prevalence was 59% (95% CI 45%, 71%) for French patient conference attendees^23^, 53% (95% CI 39%, 66%) for French inpatients^23^, and 23% (95% CI 19%, 28%) for Canadian outpatients^24,25^.

**Table 2:** Prevalence of mood and anxiety disorders.

First author	Year	Interview	Population	Current or 30-day		Lifetime	
				N cases/assessed	Prevalence (95% CI)	N cases/assessed	Prevalence (95% CI)
Mood disorders							
Major depressive disorder							
Baubet^23^	2011	MINI	French patient organization conference attendees	5/51	10% (4%, 21%)	30/51	59% (45%, 71%)
Baubet^23^	2011	MINI	French inpatients	14/49	29% (18%, 42%)	26/49	53% (39%, 66%)
Jewett/Thombs^24,25^	2014/15	CIDI	Canadian outpatients—baseline^a^	13/345	4% (2%, 6%)	79/345	23% (19%, 28%)
Jewett/Thombs^24,25^	2014/15	CIDI	Canadian outpatients—1 month follow-up	16/309	5% (3%, 8%)	–	
Jha^26^	2022	CIS-R	Indian outpatients	17/93	18% (12%, 27%)	–	
Dysthymia							
Baubet^23^	2011	MINI	Combined French patient organization conference attendees and inpatients	14/100	14% (9%, 22%)	14/100	14% (9%, 22%)
Anxiety disorders							
Any anxiety disorder							
Baubet^23^	2011	MINI	French patient organization conference attendees	25/51	49% (36%, 62%)	32/51	63% (49%, 75%)
Baubet^23^	2011	MINI	French inpatients	25/49	51% (38%, 64%)	32/49	65% (51%, 77%)
Social anxiety disorder							
Baubet^23^	2011	MINI	Combined French patient organization conference attendees and inpatients	13/100	13% (8%, 21%)	15/100	15% (9%, 23%)
Panic disorder							
Baubet^23^	2011	MINI	Combined French patient organization conference attendees and inpatients	6/100	6% (3%, 12%)	10/100	10% (6%, 17%)
Agoraphobia							
Baubet^23^	2011	MINI	Combined French patient organization conference attendees and inpatients	9/100	9% (5%, 16%)	11/100	11% (6%, 19%)
Generalized anxiety disorder							
Baubet^23^	2011	MINI	Combined French patient organization conference attendees and inpatients	13/100	13% (8%, 21%)	19/100	19% (13%, 28%)
Jha^26^	2022	CIS-R	Indian outpatients	3/93	3% (1%, 9%)	–	
Obsessive–compulsive disorder							
Baubet^23^	2011	MINI	Combined French patient organization conference attendees and inpatients	2/100	2% (1%, 7%)	2/100	2% (1%, 7%)
Jha^26^	2022	CIS-R	Indian outpatients	14/93	15% (9%, 24%)	–	
Mixed anxiety depressive disorder							
Jha^26^	2022	CIS-R	Indian outpatients	5/93	5% (2%, 12%)	–	

*CIDI* composite international diagnostic interview, *CIS-R* revised clinical interview schedule, *MINI* mini international neuropsychiatric interview.

^a^The study also reported 12-month prevalence for the baseline sample as 37/345 (11%; 95% CI 8–14%).

### Prevalence of anxiety disorders

Current or 30-day prevalence of any anxiety disorder was 49% (95% CI 36%, 62%) for French patient conference attendees and 51% (95% CI 38%, 64%) for French inpatients. Lifetime prevalence was 63% (95% CI 49%, 75%) for conference attendees and 65% (95% CI 51%, 77%) for inpatients^23^. Current or 30-day generalized anxiety disorder prevalence was 13% (95% CI 8%, 21%) for the combined French samples^23^, compared with 3% (95% CI 1%, 9%) for Indian outpatients^26^. Current or 30-day obsessive–compulsive disorder prevalence was 2% (95% CI 1%, 7%) for the combined French samples^23^ versus 15% (95% CI 9%, 24%) for Indian outpatients^26^. See Table 2^23^.

### Factors associated with mood and anxiety symptoms

Factors associated with depressive and anxiety symptoms are reported in Table 3. Of the 2 studies with at least 200 participants that examined factors associated with depressive symptoms, age^28,29^, disease subtype^28^, disease duration^28,29^, and skin scores^28,29^ were not associated with depressive symptoms, but there was some evidence of an association between higher education and lower depressive symptoms^28,29^. In addition, being married or living as married was associated with lower symptoms^28,29^. Pulmonary involvement, breathing problems, and tender joint count were associated with higher depressive symptoms^29^. The third study, which included 114 participants, assessed factors associated with both dichotomous depressive and anxiety symptoms and found no statistically significant associations between anxiety symptoms and factors assessed but statistically significant associations between depressive symptoms and disease subtype, gastrointestinal involvement, pulmonary involvement, and dyspnea^27^.

**Table 3:** Factors associated with mood or anxiety symptoms in multivariable analyses^a^.

	Depressive symptoms			Anxiety symptoms
	Faezi (2017)^27^	Kwakkenbos (2012)^28^	Thombs (2008)^29^	Faezi (2017)^27^
N	114	215	376	114
Outcome	BDI ≥ 11	CES-D score	CES-D score	Cattell ≥ 7
Unit of magnitude of association	Odds ratio (95% CI)	Non-standardized linear regression coefficient (95% CI)	Non-standardized linear regression coefficient (95% CI)	Odds ratio (95% CI)
Sociodemographic				
Age (continuous), years	0.96 (0.93, 1.23)	− 0.08 (− 0.21, 0.04)	− 0.01 (− 0.02, 0.00)	0.97 (0.96, 1.0)
Education				
Lower than high school (ref = high school or higher)	0.94 (0.52, 2.4)	–	–	1.12 (0.43, 1.89)
More than high school education (ref = high school or lower)	–	− 1.41 (− 4.32, 1.50)	− 0.46 (− 0.73, − 0.19)	–
Marital status				
Divorced (ref = single)	1.69 (0.39, 7.4)	–	–	2.23 (0.39, 7.4)
Widowed (ref = single)	3.21 (0.89, 11.6)	–	–	4.01 (0.69, 13.6)
Married (ref = single)	0.93 (0.42, 2.1)	–	–	0.93 (0.62, 2.7)
Married/cohabitating (ref = NR)	–	− 3.76 (− 7.00, − 0.52)	–	–
Married/living as married (ref = single/divorced/widowed)	–	–	− 0.54 (− 0.83, − 0.24)	–
Female (ref = male)	0.87 (0.67, 1.67)	3.95 (0.97, 6.93)	0.27 (− 0.13, 0.68)	0.77 (0.44, 1.37)
Disease variables				
Diffuse disease subtype (ref = limited disease subtype)	4.45 (2.04, 8.11)	0.95 (− 2.50, 4.40)	–	1.56 (0.65, 1.78)
Disease duration				
Disease duration (continuous)	–	0.04 (− 0.13, 0.21)	–	–
Time since onset of non-Raynaud's symptoms (continuous)	–	–	0.01 (− 0.01, 0.02)	–
Disease severity and characteristics				
Physician-rated disease severity (continuous)	–	–	0.11 (− 1.92, 2.00)	–
Hospitalization history—positive (ref = negative)	1.21 (0.31, 2.01)	–	–	1.21 (0.65, 2.71)
Modified Rodnan skin score (continuous)	–	0.22 (− 0.03, 0.47)	− 0.00 (− 0.02, 0.01)	–
Raynaud phenomenon—absent (ref = present)	0.67 (0.43, 3.55)	–	–	0.75 (0.34, 1.87)
Gastrointestinal involvement (ref = absent)	1.23 (1.16, 2.06)	–	–	0.96 (0.67, 3.66)
Number of gastrointestinal symptoms (continuous)	–	–	0.12 (0.06, 0.16)	–
Pulmonary involvement—absent (ref = present)	0.82 (0.79, 0.94)	–	–	0.46 (0.34, 1.82)
Dyspnea—absent (ref = present)	0.76 (0.34, 0.88)	–	–	2.67 (0.44, 5.29)
Breathing problems (continuous)	–	–	0.14 (0.08, 0.20)	–
Alveolitis—absent (ref = present)	–	–	–	0.49 (0.22, 3.31)
Tender joint count (continuous)	–	–	0.03 (0.00, 0.06)	–
Pulmonary hypertension—absent (ref = present)	0.59 (0.29, 1.61)	–	–	0.65 (0.15, 2.01)
Hypothyroidism—absent (ref = present)	2.79 (0.62, 4.56)	–	–	1.49 (0.29, 4.61)
Cytotoxic treatment—positive (ref = negative)	1.40 (0.78, 1.87)	–	–	1.19 (0.82, 2.56)

*BDI* beck depression inventory, *Cattell* Cattell Anxiety Self-assessment Scale, *CES-D* center for epidemiological studies-depression, *NR* not reported.

^a^Odds ratios and linear regression coefficients of some included studies were reported here in the opposite direction from the primary study to have the same reference or direction as the other included studies.

## Discussion

Our main finding was that there is limited evidence on the prevalence of mood and anxiety disorders and factors that contribute to symptoms among people with SSc. We identified only 3 studies that examined prevalence using validated diagnostic research tools, and estimates varied widely across studies. Current or 30-day MDD prevalence ranged from 4% for Canadian outpatients based on the CIDI^24,25^ to 10% among French patient conference attendees and 29% for French inpatients based on the MINI^23^ and 18% for outpatients from India based on the CIS-R^26^. Similar patterns were observed for lifetime MDD. Results for anxiety disorders were similarly inconsistent across studies. Current or 30-day generalized anxiety disorder prevalence was 13% for combined French patient organization conference attendees and inpatients based on the MINI^23^, compared with only 3% for Indian outpatients based on the CIS-R^26^. On the other hand, only 2% of combined French participants were classified as having current or 30-day obsessive-compulsive disorder, compared to 15% of Indian outpatients.

The large differences in prevalence estimates across studies could be due in part to the small sample sizes and imprecise estimates, but they may also be because studies differed by country, setting, disease characteristics, and the assessment tools used to classify disorders. Indeed, there are important differences in commonly used classification methods. Semi-structured interviews (e.g., Structured Clinical Interview for DSM; SCID^13^) are designed to most closely replicate diagnostic standards and procedures; they are intended for administration by trained professionals with diagnostic experience, and evaluators can interject queries and use their clinical judgment to determine whether symptoms are present and significant^30–32^. Fully structured interviews, which were used in the studies included in our systematic review (e.g., CIDI, MINI, CIS-R), in contrast, are designed for lay-interviewer administration to reduce the cost of clinician-administered interviews. They are completely scripted, and evaluators cannot provide additional explanations or rephrase questions; minimal judgment is involved. They are intended to maximize reliability but may reduce validity^33^. A synthesis of results from 3 individual participant data meta-analyses (212 studies, 69,405 participants)^34^ found that compared to a semi-structured diagnostic interview, a typical fully structured interview, the CIDI, was more likely to classify individuals with mild depressive symptoms and less likely to classify individuals with more severe symptoms compared with the SCID. The MINI, which is a very brief fully structured interview designed to be over-inclusive^35,36^, overestimated depression prevalence substantially across the spectrum of symptom severity. In our systematic review, MDD prevalence was almost 3 times higher for French patient organization conference attendees based on the MINI than for Canadian outpatients based on the CIDI^23–25^. Prevalence of mood and anxiety disorders for Indian outpatients based on the CIS-R was also high overall^26^, but the CIS-R is less commonly used, and its performance has not been investigated compared to semi-structured interviews.

We identified 2 systematic reviews that evaluated prevalence of major depression or anxiety disorders based on DSM or ICD criteria, 1 in rheumatoid arthritis^37^ and 1 in systemic lupus erythematosus^38^. However, neither required that a validated diagnostic interview was used as an inclusion criterion, and few included studies did this, which limits interpretability and the ability to compare results to those in SSc.

Three studies (N = 114–376) assessed sociodemographic and disease-related factors associated with depressive symptoms^27–29^, and 1 study (N = 114) also assessed factors associated with anxiety symptoms^27^. None of the reviewed studies included large enough samples to draw strong conclusions. In the 2 studies with at least 200 participants, being married or living as married was associated with lower depressive symptoms^28,29^. Among disease-related variables, gastrointestinal symptoms, breathing problems, and tender joint count were associated with higher depressive symptoms^29^, while disease subtype^28^, disease duration^28,29^, and disease severity^28,29^ were not associated. These factors, however, were often measured crudely in studies (e.g., by self-report) or were not measured consistently between studies.

Future studies should examine prevalence of mood and anxiety disorders in SSc in large, representative samples. Ideally, they would be done using best-practice semi-structured interviews or a commonly used fully structured interview, such as the CIDI, to facilitate comparison. These interviews are resource-intensive, but study designs have been proposed, such as two-stage sampling, that can reduce resource requirements substantially and still generate valid and reasonably precise estimates^12^. Similarly, larger studies with representative samples using robust, high-quality multivariate assessment of factors are needed. Importantly, such studies should exclude other concurrently measured mental health variables or other self-reported outcomes for which directionality with depressive or anxiety symptoms is unclear (e.g., pain, fatigue, self-efficacy).

Although estimates differed across studies, prevalence of mood and anxiety disorders is certainly high in SSc. The Canadian study, for instance, which reported the lowest MDD prevalence pointed out that prevalence was approximately double that of the Canadian general population^25^. Health care professionals should be alert to clinical cues that could suggest depression or anxiety and ask appropriate questions and follow-up with assessment or referral for assessment, as appropriate. In addition, information about mental health may be provided in clinics, including information on self-help programs or peer support that may be available as a first step in providing psychosocial support. Ideally, psychological interventions for depressive and anxiety symptoms would be integrated into interdisciplinary care^39^. Depression and anxiety screening has been recommended in rheumatoid arthritis, psoriasis, and psoriatic arthritis^40,41^. However, randomized trials have evaluated the effects of screening for depression in postpartum women, patients with osteoarthritis, patients with post-acute coronary syndrome, and post-deployment military personnel, and none have found that depression screening improved mental health outcomes^39^; to date, there are no trials of screening for anxiety disorders. Mental health screening would require referral of large numbers of patients for psychiatric assessment, and some patients would be treated. But, based on trials conducted in other medical conditions, this would not likely improve mental health.

Strengths of our systematic review include the use rigorous best-practice methods consistent with Cochrane recommendations; searching multiple databases; not restricting inclusion by language; and the recency of our searches and ability to update rapidly as evidence emerges via our living systematic review approach. There are also limitations that suggest that caution should be used in interpreting results. All included studies had sample sizes < 400 and had limitations related to study sampling frames and recruitment methods. Included studies that assessed prevalence of mood or anxiety disorders used fully structured diagnostic interviews, which are are intended to maximize reliability but may reduce validity compared to semi-structured interviews, which most closely replicate actual diagnostic procedures^30,33^.

## Summary

We reviewed primary studies on the prevalence of mood and anxiety disorders and factors associated with symptoms. We found that the prevalence of mood and anxiety disorders appears to be high in SSc, but estimates vary widely depending on the sample characteristics and instrument used for classification. Future research that uses semi-structured interview methods or commonly used and well-validated fully structured interviews and that include large numbers of representative patients are needed. Similarly, large studies of representative samples that use validated symptom measurements and high-quality, robust multivariate factor assessment are needed. This is the first report from our living systematic review of the prevalence of mood and anxiety disorders and factors associated with symptoms in people with SSc. We will continue to update results as they become available via our living systematic review approach, and ongoing dissemination of results will be facilitated via posting to the project website (https://www.spinsclero.com/living-systematic-reviews/depression-and-anxiety-in-scleroderma).

## Supplementary Information

Supplementary Information.

## Data Availability

All data generated or analysed during this study are included in this published article.
