# Supplementary material for: Results from a living systematic review of the prevalence of mood and anxiety disorders and factors associated with symptoms in systemic sclerosis
Source: Sci Rep. 2023 Mar 30;13:5181. doi: 10.1038/s41598-023-31919-8 (PMC10063612; doi:10.1038/s41598-023-31919-8)
Supplement: Supplementary file 1 — Supplementary Information. [file 41598_2023_31919_MOESM1_ESM.docx]

**APPENDIX 1. Search strategy**

**Database(s): Ovid MEDLINE(R) ALL 1946 to March 1, 2023**

Search Strategy:

# Searches

1 Depression

2 exp Depressive Disorder

3 Bipolar Disorder

4 (depression or depressive or social phobia or agoraphobia or dysthymia or bipolar or obsessive compulsive or OCD or anxiety or ((mood or panic or phobic or affective) adj3 disorder*)).tw,kf.

5 exp Anxiety Disorders

6 Mood Disorders

7 exp Scleroderma, Systemic/ or (scleroderma* or systemic sclerosis or CRESTsyndrome*).ti,ab,kf.

8 or/1-6

9 7 and 8

**Database(s): Embase Classic+Embase 1947 to 2023 March 1**

Search Strategy:

# Searches

1 exp *depression

2 exp *bipolar disorder

3 exp *anxiety disorder

4 *anxiety

5 *mood disorder

6 (depression or depressive or social phobia or agoraphobia or dysthymia or bipolar or obsessive compulsive or OCD or anxiety or ((mood or panic or phobic or affective) adj3 disorder*)).tw,kw.

7 1 or 2 or 3 or 4 or 5 or 6

8 exp *scleroderma/ or (scleroderma* or systemic sclerosis or CREST syndrome*).tw,kw.

9 7 and 8

**Database(s): APA PsycInfo 1967 to March Week 1 2023**

Search Strategy:

# Searches

1 exp major depression

2 exp bipolar disorder

3 anxiety/ or social anxiety

4 exp Anxiety Disorders

5 affective disorders

6 (depression or depressive or social phobia or agoraphobia or dysthymia or bipolar or obsessive compulsive or OCD or anxiety or ((affective or mood or panic or phobic) adj3 disorder*)).tw,id.

7 1 or 2 or 3 or 4 or 5 or 6

8 (scleroderma* or systemic sclerosis or CREST syndrome*).tw,id. or skin disorders/ or "Sclerosis (Nervous System)"/

9 7 and 8

**CINAHL**

| **Search ID#** | **Search Terms** | **Results** |
| --- | --- | --- |
| S8 | S6 AND S7 |  |
| S7 | ( TI ( (scleroderma* or systemic sclerosis or CREST syndrome*) ) OR AB ( (scleroderma* or systemic sclerosis or CREST syndrome*) ) ) OR (MH "Scleroderma, Systemic+") |  |
| S6 | S1 OR S2 OR S3 OR S4 OR S5 |  |
| S5 | (MH "Affective Disorders") |  |
| S4 | (MH "Anxiety Disorders+") OR (MH "Anxiety") |  |
| S3 | TI ( depression or depressive or social phobia or agoraphobia or dysthymia or bipolar or obsessive compulsive or OCD or anxiety or ((mood or panic or phobic or affective) adj3 disorder*)) ) OR AB ( depression or depressive or social phobia or agoraphobia or dysthymia or bipolar or obsessive compulsive or OCD or anxiety or ((mood or panic or phobic or affective) adj3 disorder*)) ) |  |
| S2 | (MH "Bipolar Disorder+") | Display |
| S1 | (MH "Depression+") | Display |

**Cochrane Central:**

(depression or depressive or social phobia or agoraphobia or dysthymia or bipolar or obsessive compulsive or OCD or anxiety or ((mood or panic or phobic or affective) NEAR/3 disorder*)):ti,ab,kw

AND

(scleroderma* or systemic sclerosis or CREST syndrome*):ti,ab,kw

**APPENDIX 2. Inclusion and exclusion coding manual**

***Title/Abstract Coding Manual***

| PREVALENCE CODING CRITERIA:  **No: not primary data.** If it is clear from the title or abstract that the article is a case study/report, letter to the editor, editorial, systematic review, or meta-analysis, it will be excluded. Articles from conferences or symposia will be included.  **No: no participants with SSc.** If it is clear from the title or abstract that the study sample does not consist of participants with SSc, with a sample size of at least 50 persons, it will be excluded. Studies reporting on different populations that do not report outcomes separately by participant group will be excluded if < 80% of patients have SSc; studies that report outcomes separately may be included if they meet all other requirements.  **No: no mood or anxiety classification.** If it is clear from the title or abstract that participants are not assessed for diagnostic status using a validated diagnostic interview based on the Diagnostic and Statistical Manual of Mental Disorders (DSM) or the International Statistical Classification of Diseases (ICD), it will be excluded. Studies that use other methods (e.g., self-report questionnaires, rating scales, unstructured diagnosis, chart records) will be excluded.  **No: does not assess prevalence.** If it is clear from the title or abstract that the study does not report on the prevalence of mood or anxiety disorders among participants with SSc, it will be excluded.  **Yes: study eligible for inclusion in full-text review.** | FACTORS CODING CRITERIA:  **No: not primary data.** If it is clear from the title or abstract that the article is a case study/report, letter to the editor, editorial, systematic review, or meta-analysis, it will be excluded. Articles from conferences or symposia will be included.  **No: no participants with SSc.** If it is clear from the title or abstract that the study sample does not consist of participants with SSc, with a sample size of at least 100 persons, it will be excluded. Studies reporting on different populations that do not report outcomes separately by participant group will be excluded if < 80% of patients have SSc; studies that report outcomes separately may be included if they meet all other requirements.  **No: no valid mood or anxiety assessment.** If it is clear from the title or abstract that the study does not include a validated diagnostic interview or a validated mental health scale (e.g., anxiety, depression, stress, loneliness, fear, anger, grief, other related), then it will be excluded. Studies that use assessments based on single self-report items will be excluded.  **No: factors assessed do not relate to mood and anxiety symptoms.** If it is clear from the title or abstract that the study does not report on multivariable evaluations of variables associated with mood and anxiety symptoms or diagnoses as outcomes, it will be excluded. If factors in a multivariate model include other concurrently measured mental health variables or other self-reported outcomes for which directionality is unclear (e.g., pain, fatigue), the study will be excluded. If the study uses outcome symptom levels as an eligibility criterion, it will be excluded.  **Yes: study eligible for inclusion in full-text review.** |
| --- | --- |

***Full Text Inclusion/Exclusion Coding Manual***

| PREVALENCE CODING CRITERIA:  **No: not primary data.** If the article is a case study/report, letter to the editor, editorial, systematic review, or meta-analysis, it will be excluded. Articles from conferences or symposia will be included.  **No: no participants with SSc.** If the study sample does not consist of participants with SSc, with a sample size of at least 50 persons, it will be excluded. Studies reporting on different populations that do not report outcomes separately by participant group will be excluded if < 80% of patients have SSc; studies that report outcomes separately may be included if they meet all other requirements.  **No: no mood or anxiety classification.** If participants were not assessed for diagnostic status using a validated diagnostic interview based on the Diagnostic and Statistical Manual of Mental Disorders (DSM) or the International Statistical Classification of Diseases (ICD), it will be excluded. Studies that use other methods (e.g., self-report questionnaires, rating scales, unstructured diagnosis, chart records) will be excluded.  **No: does not assess prevalence.** If the study does not report on the prevalence of mood or anxiety disorders among participants with SSc, it will be excluded.  **Yes: study eligible for inclusion in the systematic review.** | FACTORS CODING CRITERIA:  **No: not primary data.** If the article is a case study/report, letter to the editor, editorial, systematic review, or meta-analysis, it will be excluded. Articles from conferences or symposia will be included.    **No: no participants with SSc.** If the study sample does not consist of participants with SSc, with a sample size of at least 100 persons, it will be excluded. Studies reporting on different populations that do not report outcomes separately by participant group will be excluded if < 80% of patients have SSc; studies that report outcomes separately may be included if they meet all other requirements.    **No: no valid mood or anxiety assessment.** If the study does not include a validated diagnostic interview or a validated mental health scale (e.g., anxiety, depression, stress, loneliness, fear, anger, grief, other related), then it will be excluded. Studies that use assessments based on single self-report items will be excluded.    **No: factors assessed do not relate to mood and anxiety symptoms.** If the study does not report on multivariable evaluations of variables associated with mood and anxiety symptoms or diagnoses as outcomes, it will be excluded. If factors in a multivariate model include other concurrently measured mental health variables or other self-reported outcomes for which directionality is unclear (e.g., pain, fatigue), the study will be excluded. If the study uses outcome symptom levels as an eligibility criterion, it will be excluded.  **Yes: study eligible for inclusion in the systematic review.** |
| --- | --- |

**APPENDIX 3. Variables Included in Data Extraction Form**

***Data Extraction – Study Variables***

1. Authors (first author last name)
2. Publication year (use "preliminary version" instead of year if not in final version)
3. Journal

Note: Please enter full journal name or full preprint server name (e.g., “Open Science Framework", “MedRxiv”)

1. Funding Source

Source of financial support: In addition to reading the article, please do a “apple/control F” search for key word stems, including “financ”, “conflict”, “compet”, “fund”, “grant”, "spon" "discl"

Response from radio options:

Industry

Combined industry and non-industry

Non-industry

No study funding

Not reported

1. Single or Multiple Eligible Time Points?

Some studies may report eligible analyses or outcomes at more than one time point.

Response from radio options:

Single

Multiple

How many, if multiple?

1. Country(ies) of participants

Response from radio options:

Country name (from list of all countries)

Unspecified

Unknown

More than one country(specify)

1. Dates of data collection

Note: Use format MM/YYYY-MM/YYYY (if only years provided, use YYYY-YYYY)

1. Number of participants

Please indicate the total sample size of SSc participants within the study.

If multiple analyses used with differing number of participants: n= x-y (range)

1. What is the format of this article/study?

Full-text, abstract, pre-print, etc.

1. Recruitment method

Patient organizations: participants in the study were invited to join via established organization (e.g., Scleroderma Canada)

Participant pools: the sample for this study was taken from a larger cohort (e.g., SPIN-CHAT trial participants mostly came from larger SPIN cohort)

Clinical sampling: participants were taken from hospital/clinic settings

1. Eligibility Criteria

Please provide the main eligibility criteria for participant entry into the study, one criterion per line.

E.g. Adults above age 25, confirmed diagnosis of SSc, Severity of disease

***Data Extraction – Characteristics***

1. SSc characteristics of participants: AGE

Provide: mean (standard deviation).

Alternatively, if not reported, provide an alternative, such as the median or percentage in certain age groups. Specify the measure being provided if mean (SD) not given. Eg. a breakdown of ages based on demographics (25% ages 18-30, 50% 31-59, 25% 60+), median, range

Please round to report with no decimals (e.g., 75.) If not provided, note, "NR".

1. SSc characteristics of participants: SEX

Indicate percent of female/women (% F/W)

If unclear, indicate with "Unknown"

Please round to report with no decimals (e.g., "14%"). If not provided, note, "NR".

1. SSc characteristics of participants: DIAGNOSIS

Indicate how study reported time since diagnosis (time since onset of symptoms, time since diagnosis, time since first non-Raynaud's symptoms).

An unspecified index event is where reporting of time since X is unknown (eg. mean disease duration).

Further, indicate (mean, standard deviation) if provided.

Please round to report with no decimals

1. SSc characteristics of participants: Type of SSc

Indicate how many individuals (per cent, n, etc) presented with diffuse, limited, sine, unknown SSc. If this information was not reported, indicate in textbox as "n/s".

Occasionally, limited/sine will be presented together - pick "limited/sine" but only use if this is reported.

Please round to report with no decimals.

***Data Extraction – Outcome***

Which research question is this study eligible for?

1. Prevalence of mood and anxiety disorders.
2. Factors associated with symptoms of mood and anxiety disorders.
3. Diagnostic interview used (Choose all that apply)

Response from radio options:

Structured interview

Non structured interview

Semi structured interview

Other (specify)

Note: Diagnostic status assessed using a validated diagnostic interview based on the Diagnostic and Statistical Manual of Mental Disorders (DSM) or the International Statistical Classification of Diseases (ICD)

1. Type of disorder - Mood Disorder

Specify how many individuals (per cent, n, etc).

1. Any Mood Disorder (unspecified)

Prevalence Period: Checkbox

1. Current
2. N assessed (NR if not reported)
3. N cases (NR if not reported)
4. Prevalence in percent (1 decimal) (NR if not reported)
5. 30-day period
6. N assessed (NR if not reported)
7. N cases (NR if not reported)
8. Prevalence in percent (1 decimal) (NR if not reported)
9. 12-month period
10. N assessed (NR if not reported)
11. N cases (NR if not reported)
12. Prevalence in percent (1 decimal) (NR if not reported)
13. Lifetime
14. N assessed (NR if not reported)
15. N cases (NR if not reported)
16. Prevalence in percent (1 decimal) (NR if not reported)
17. Other (with text box to explain; include incidence here)
18. N assessed (NR if not reported)
19. N cases (NR if not reported)
20. Prevalence in percent (1 decimal) (NR if not reported)
21. Major Depressive Disorder

Prevalence Period: Checkbox

1. Current
2. N assessed (NR if not reported)
3. N cases (NR if not reported)
4. Prevalence in percent (1 decimal) (NR if not reported)
5. 30-day period
6. N assessed (NR if not reported)
7. N cases (NR if not reported)
8. Prevalence in percent (1 decimal) (NR if not reported)
9. 12-month period
10. N assessed (NR if not reported)
11. N cases (NR if not reported)
12. Prevalence in percent (1 decimal) (NR if not reported)
13. Lifetime
14. N assessed (NR if not reported)
15. N cases (NR if not reported)
16. Prevalence in percent (1 decimal) (NR if not reported)
17. Other (with text box to explain; include incidence here)
18. N assessed (NR if not reported)
19. N cases (NR if not reported)
20. Prevalence in percent (1 decimal) (NR if not reported)
21. Dysthymia

Prevalence Period: Checkbox

1. Current
2. N assessed (NR if not reported)
3. N cases (NR if not reported)
4. Prevalence in percent (1 decimal) (NR if not reported)
5. 30-day period
6. N assessed (NR if not reported)
7. N cases (NR if not reported)
8. Prevalence in percent (1 decimal) (NR if not reported)
9. 12-month period
10. N assessed (NR if not reported)
11. N cases (NR if not reported)
12. Prevalence in percent (1 decimal) (NR if not reported)
13. Lifetime
14. N assessed (NR if not reported)
15. N cases (NR if not reported)
16. Prevalence in percent (1 decimal) (NR if not reported)
17. Other (with text box to explain; include incidence here)
18. N assessed (NR if not reported)
19. N cases (NR if not reported)
20. Prevalence in percent (1 decimal) (NR if not reported)
21. Persistent Depressive Disorder

Prevalence Period: Checkbox

1. Current
2. N assessed (NR if not reported)
3. N cases (NR if not reported)
4. Prevalence in percent (1 decimal) (NR if not reported)
5. 30-day period
6. N assessed (NR if not reported)
7. N cases (NR if not reported)
8. Prevalence in percent (1 decimal) (NR if not reported)
9. 12-month period
10. N assessed (NR if not reported)
11. N cases (NR if not reported)
12. Prevalence in percent (1 decimal) (NR if not reported)
13. Lifetime
14. N assessed (NR if not reported)
15. N cases (NR if not reported)
16. Prevalence in percent (1 decimal) (NR if not reported)
17. Other (with text box to explain; include incidence here)
18. N assessed (NR if not reported)
19. N cases (NR if not reported)
20. Prevalence in percent (1 decimal) (NR if not reported)
21. Bipolar Disorder

Prevalence Period: Checkbox

1. Current
2. N assessed (NR if not reported)
3. N cases (NR if not reported)
4. Prevalence in percent (1 decimal) (NR if not reported)
5. 30-day period
6. N assessed (NR if not reported)
7. N cases (NR if not reported)
8. Prevalence in percent (1 decimal) (NR if not reported)
9. 12-month period
10. N assessed (NR if not reported)
11. N cases (NR if not reported)
12. Prevalence in percent (1 decimal) (NR if not reported)
13. Lifetime
14. N assessed (NR if not reported)
15. N cases (NR if not reported)
16. Prevalence in percent (1 decimal) (NR if not reported)
17. Other (with text box to explain; include incidence here)
18. N assessed (NR if not reported)
19. N cases (NR if not reported)
20. Prevalence in percent (1 decimal) (NR if not reported)
21. Other (please indicate)

Prevalence Period: Checkbox

1. Current
2. N assessed (NR if not reported)
3. N cases (NR if not reported)
4. Prevalence in percent (1 decimal) (NR if not reported)
5. 30-day period
6. N assessed (NR if not reported)
7. N cases (NR if not reported)
8. Prevalence in percent (1 decimal) (NR if not reported)
9. 12-month period
10. N assessed (NR if not reported)
11. N cases (NR if not reported)
12. Prevalence in percent (1 decimal) (NR if not reported)
13. Lifetime
14. N assessed (NR if not reported)
15. N cases (NR if not reported)
16. Prevalence in percent (1 decimal) (NR if not reported)
17. Other (with text box to explain; include incidence here)
18. N assessed (NR if not reported)
19. N cases (NR if not reported)
20. Prevalence in percent (1 decimal) (NR if not reported)
21. Type of disorder - Anxiety Disorder

Specify how many individuals (per cent, n, etc).

1. Any Anxiety Disorder (unspecified)

Prevalence Period: Checkbox

1. Current
2. N assessed (NR if not reported)
3. N cases (NR if not reported)
4. Prevalence in percent (1 decimal) (NR if not reported)
5. 30-day period
6. N assessed (NR if not reported)
7. N cases (NR if not reported)
8. Prevalence in percent (1 decimal) (NR if not reported)
9. 12-month period
10. N assessed (NR if not reported)
11. N cases (NR if not reported)
12. Prevalence in percent (1 decimal) (NR if not reported)
13. Lifetime
14. N assessed (NR if not reported)
15. N cases (NR if not reported)
16. Prevalence in percent (1 decimal) (NR if not reported)
17. Other (with text box to explain; include incidence here)
18. N assessed (NR if not reported)
19. N cases (NR if not reported)
20. Prevalence in percent (1 decimal) (NR if not reported)
21. Social Phobia (or Social Anxiety Disorder)

Prevalence Period: Checkbox

1. Current
2. N assessed (NR if not reported)
3. N cases (NR if not reported)
4. Prevalence in percent (1 decimal) (NR if not reported)
5. 30-day period
6. N assessed (NR if not reported)
7. N cases (NR if not reported)
8. Prevalence in percent (1 decimal) (NR if not reported)
9. 12-month period
10. N assessed (NR if not reported)
11. N cases (NR if not reported)
12. Prevalence in percent (1 decimal) (NR if not reported)
13. Lifetime
14. N assessed (NR if not reported)
15. N cases (NR if not reported)
16. Prevalence in percent (1 decimal) (NR if not reported)
17. Other (with text box to explain; include incidence here)
18. N assessed (NR if not reported)
19. N cases (NR if not reported)
20. Prevalence in percent (1 decimal) (NR if not reported)
21. Panic Disorder

Prevalence Period: Checkbox

1. Current
2. N assessed (NR if not reported)
3. N cases (NR if not reported)
4. Prevalence in percent (1 decimal) (NR if not reported)
5. 30-day period
6. N assessed (NR if not reported)
7. N cases (NR if not reported)
8. Prevalence in percent (1 decimal) (NR if not reported)
9. 12-month period
10. N assessed (NR if not reported)
11. N cases (NR if not reported)
12. Prevalence in percent (1 decimal) (NR if not reported)
13. Lifetime
14. N assessed (NR if not reported)
15. N cases (NR if not reported)
16. Prevalence in percent (1 decimal) (NR if not reported)
17. Other (with text box to explain; include incidence here)
18. N assessed (NR if not reported)
19. N cases (NR if not reported)
20. Prevalence in percent (1 decimal) (NR if not reported)
21. Agoraphobia

Prevalence Period: Checkbox

1. Current
2. N assessed (NR if not reported)
3. N cases (NR if not reported)
4. Prevalence in percent (1 decimal) (NR if not reported)
5. 30-day period
6. N assessed (NR if not reported)
7. N cases (NR if not reported)
8. Prevalence in percent (1 decimal) (NR if not reported)
9. 12-month period
10. N assessed (NR if not reported)
11. N cases (NR if not reported)
12. Prevalence in percent (1 decimal) (NR if not reported)
13. Lifetime
14. N assessed (NR if not reported)
15. N cases (NR if not reported)
16. Prevalence in percent (1 decimal) (NR if not reported)
17. Other (with text box to explain; include incidence here)
18. N assessed (NR if not reported)
19. N cases (NR if not reported)
20. Prevalence in percent (1 decimal) (NR if not reported)
21. Generalized Anxiety Disorder

Prevalence Period: Checkbox

1. Current
2. N assessed (NR if not reported)
3. N cases (NR if not reported)
4. Prevalence in percent (1 decimal) (NR if not reported)
5. 30-day period
6. N assessed (NR if not reported)
7. N cases (NR if not reported)
8. Prevalence in percent (1 decimal) (NR if not reported)
9. 12-month period
10. N assessed (NR if not reported)
11. N cases (NR if not reported)
12. Prevalence in percent (1 decimal) (NR if not reported)
13. Lifetime
14. N assessed (NR if not reported)
15. N cases (NR if not reported)
16. Prevalence in percent (1 decimal) (NR if not reported)
17. Other (with text box to explain; include incidence here)
18. N assessed (NR if not reported)
19. N cases (NR if not reported)
20. Prevalence in percent (1 decimal) (NR if not reported)
21. Other (please specify)

Prevalence Period: Checkbox

1. Current
2. N assessed (NR if not reported)
3. N cases (NR if not reported)
4. Prevalence in percent (1 decimal) (NR if not reported)
5. 30-day period
6. N assessed (NR if not reported)
7. N cases (NR if not reported)
8. Prevalence in percent (1 decimal) (NR if not reported)
9. 12-month period
10. N assessed (NR if not reported)
11. N cases (NR if not reported)
12. Prevalence in percent (1 decimal) (NR if not reported)
13. Lifetime
14. N assessed (NR if not reported)
15. N cases (NR if not reported)
16. Prevalence in percent (1 decimal) (NR if not reported)
17. Other (with text box to explain; include incidence here)
18. N assessed (NR if not reported)
19. N cases (NR if not reported)
20. Prevalence in percent (1 decimal) (NR if not reported)
21. Additional notes
22. Measure

Note: e.g. GHQ. Please include cut-off scores for logistic regression. If cut-off isn't reported, write NR. If multiple analyses are conducted with the same outcome measure, extract for the fully adjusted model.

1. Type of analysis

Response from radio options:

Multiple linear regression

Multiple logistic regression

Other-please specify

1. Note: Please list all variables analyzed. If some variables aren't reported in the table, list them here and write "NS" for not significant. Link to linear regression calculator. Type: Label
2. Associated factors included in multivariable analysis

Note: please list one factor per row. Type: Table Subform, Available form: Factors Table

1. Notes: Please add any notes on how you extracted data, particularly if it was not straightforward. Type: text
2. Factor

If applicable, please use this format: Female (ref=Male), Undergraduate education (ref=High School). Type: text

1. Unit of magnitude of association

e.g., odds ratio, raw linear regression coefficient, standardized linear regression coefficient.

For linear regression, prioritize standardized coefficient and CI if available. Use calculator for unreported values. Report unstandardized if not. Type: text

1. Magnitude of association

Report value, e.g., 1.82, 2 decimals. Type: text

1. 95% CI

If provided, report with 2 decimals e.g. 1.82

Use format (1.23,4.56). Type: text

1. P Value

Type: text

**APPENDIX 4: Checklist for studies reporting on prevalence of mood and anxiety disorders: adapted from JBI critical appraisal checklist for prevalence studies**

1. **Was the sample frame appropriate to address the target population?**

This question relies upon knowledge of the broader characteristics of the population of interest and the geographical area. The term “target population” does not mean that we would be looking for every individual with scleroderma (for example). **Instead, give consideration to specific population characteristics in the study, including age range, gender, morbidities, and other potentially influential factors.** For example, a sample frame may not be appropriate to address the target population if a certain group has been used (such as individuals with scleroderma who were aged at least 60 years) and the results then inferred to the target population (i.e., individuals with scleroderma of any age). A sample frame may be appropriate when it includes almost all the members of the target population (i.e., a census or a complete list of participants or complete registry data).

| Yes: The sampling frame was a true or close representation of the target population.  No: The sampling frame was NOT a true or close representation of the target population.  Unclear: Not enough information provided to determine. | Examples:   - The target population was the population of individuals with scleroderma of any age, sex, and disease severity, and eligible participants included all individuals with scleroderma of any age, sex, and disease severity. The answer is: Yes. - The target population was the population of individuals with scleroderma of any age, sex, and disease severity, and eligible participants included only individuals with scleroderma who were at least 60 years of age. The answer is: No. |
| --- | --- |

1. **Were study participants recruited in an appropriate way?**

Studies may report random sampling from a population, and the methods section should report how sampling was performed. Random sampling from a defined subset of the population (sample frame) should be employed in most cases, however, random probabilistic sampling is not needed when everyone in the sampling frame will be included/ analysed. For example, reporting on all the data from a good census is appropriate as a good census will identify everybody. When using cluster sampling, such as a random sample of hospitals within a region, the methods need to be clearly stated. Convenience samples, such as samples recruited from existing organizations of nurses is not considered to provide a representative sample of the base population.

| Yes: A census was undertaken, OR, some form of random selection was used to select the sample (e.g. simple random sampling, stratified random sampling, cluster sampling, systematic sampling).  No: A census was NOT undertaken, AND some form of random selection was NOT used to select the sample.  Unclear: Not enough information provided to determine. | A census collects information from every unit in the sampling frame. In a survey, only part of the sampling frame is sampled. In these instances, random selection of the sample helps minimize study bias. Examples:   - The target population was the population of individuals with scleroderma of any age and sex, and consecutive sampling of all patients or a random sample was done. The answer is: Yes. - The target population was the population of individuals with scleroderma of any age and sex, and a convenience sample in a clinical setting was done or a Facebook advertisement was placed on scleroderma pages to encourage people to go online to respond to the survey: No. |
| --- | --- |

1. **Was the sample size adequate?**

The larger the sample, the narrower the confidence interval around the point estimate will be, making the results more precise. An adequate sample size is important to ensure good precision of the final estimate. Ideally, we are looking for evidence that the authors conducted a sample size calculation to determine an adequate sample size. For conditions with small proportions of change, a larger sample size is needed. Sometimes, the study will be large enough (as in large national surveys) whereby a sample size calculation is not required. In these cases, sample size can be considered adequate.

| Yes: There is evidence that the authors conducted and properly carried out a sample size calculation to determine an adequate sample size OR the study was large enough (e.g., a large national survey) whereby a sample size calculation is not required. In these cases, sample size can be considered adequate. If a sample size calculation was not done, and at least 200 participants are included for **continuous** outcomes and 250 for **proportions**, this is considered Yes.  No: The authors did not reach their intended sample size, or no sample size calculation is provided and there are < 100 participants for continuous outcomes, or < 125 for proportions.  Unclear: No sample size calculation is provided, and between 100-199 participants are included for continuous outcomes or between 125-249 for proportions. | Examples:   - The target population was determined prior to beginning the study and an adequate number of participants were recruited: Yes. - The target sample size could not be recruited and fewer participants were enrolled than deemed necessary, or the authors did not include a sample size justification and fewer than 100 participants were included. The answer is: No. |
| --- | --- |

1. **Were the study subjects and setting described in detail?**

Mental health disorders may have a different prevalence across geographic regions and populations (e.g., Women vs. Men, sociodemographic variables between countries). The study sample should be described in sufficient detail so that other researchers can determine if it is comparable to the population of interest to them.

| Yes: Data included age, sex, at least 1 socioeconomic indicator (e.g., income, education, work status), at least one clinical variable (e.g., disease severity, scleroderma sub-type), and the setting (e.g., type of health care institution).  No: The minimum sociodemographic variables, clinical variables, and setting were not been reported.  Unclear: Not stated. | Examples:   - Participants were an average age of 30.0 years (SD=10.0), mostly female (80%), 50% had diffuse scleroderma, and were recruited from an outpatient clinic. Yes. - Participants were an average age of 30.0 years (SD=10.0) and most were female (80%). No. |
| --- | --- |

1. **Was the data analysis conducted with sufficient coverage of the identified sample?**

Coverage bias can occur when not all subgroups of the identified sample respond at the same rate. For instance, you may have a very high response rate overall for your study, but the response rate for a certain subgroup (i.e., older adults) may be quite low.

| Yes: The overall response rate or response rate for intended subgroups was ≥ 75%, OR, an analysis was performed that showed no substantive differences in relevant demographic characteristics between responders and non-responders within a subgroup, AND, there’s no indication that important subgroups were underrepresented (i.e., no indication of coverage bias).  No: The overall response rate or response rate for subgroups was <75%, and if any analysis comparing responders and non-responders was done, it showed a meaningful difference in relevant demographic characteristics between responders and non-responders, OR, there is concern that certain important groups were underrepresented in sampling (i.e., some indication of coverage bias).  Unclear: Not enough information provided to determine. | Examples:   - The overall response rate was 78% and there is no indication that important subgroups were underrepresented. The answer is: Yes. - The overall response rate was 78% but there is there is concern that only 20% had diffuse disease, which is far below typical levels. The answer is: No. - The overall response rate was 50%. The answer is: No. |
| --- | --- |

1. **Were valid methods used for the identification of the condition?**

This item considers measurement or classification bias for the mental health outcome. Research has shown that semi-structured diagnostic interviews (e.g., the Structured Clinical Interview for DSM Disorders; SCID) most closely replicate the diagnostic process as described in the Diagnostic and Statistical Manual of Mental Disorders (DSM) and the International Classification of Disease (ICD) manuals, whereas most fully structured diagnostic interviews, including the Mini-International Neuropsychiatric Interview (MINI), can lead to misclassification; results remain inconclusive for the Composite International Diagnostic Interview (CIDI) (Wu et al., 2020, *J Psychosom Res*).

| Yes: The study instrument had been shown to have reliability and validity, e.g., test-retest, piloting, validation in a previous study, etc.  No: The study instrument had NOT been shown to have reliability or validity.  Unclear. | Examples   - The authors used a semi-structured diagnostic interview (e.g., the SCID), which has previously been found to have validity and reliability. The answer is: Yes. - The authors used the MINI, which has previously been found to lack validity or reliability, or a fully structured diagnostic interview. The answer is: No. - The authors used an unstructured interview and did not test this for validity or reliability. The answer is: No. - The authors used a diagnostic interview with mixed results for validity and reliability (e.g., the CIDI). The answer is: Unclear. |
| --- | --- |

1. **Was the condition measured in a standard, reliable way for all participants?**

Considerable judgment is required to determine the presence of mental health outcomes. Having established the validity of the outcome measurement instrument (see item 6 of this scale), it is important to establish how the measurement was conducted. This item considers how the outcome variable was collected, considering if the administrators had an appropriate level of education, clinical or research experience, or level of responsibility to assess an outcome.

| Yes: All self-report data were collected directly from the subjects. Any clinical interview data includes at least information about the interviewers’ level of education or training received. The same mode of data collection was used for all subjects. All aspects of this question must be present (where relevant).  No: In some instances, data were collected from a proxy (e.g., a spouse). The qualifications of clinical interviewers are not reported or not appropriate. The same mode of data collection was NOT used for all subjects. If any aspects of this item are absent, it is NO.  Unclear: Not stated. | Examples:   - All participants were interviewed by a trained psychiatrist about their mental health or were interviewed by a research coordinator with qualifications and training described. The answer is: Yes. - Participants were interviewed by an individual whose qualifications, experience, and training were not described or were not appropriate (e.g., undergraduate students administered SCID modules). The answer is: No. |  |
| --- | --- | --- |
| **Note: Semi-structured diagnostic interviews require appropriately trained individuals who are qualified and can provide diagnoses outside the context of such an interview (e.g., psychologist, psychiatrist, clinical social workers). Fully structured diagnostic interviews, however, could be conducted by appropriately trained undergraduate or graduate students or research assistants.** | | |

1. **Was there appropriate statistical analysis?**

The numerator and denominator should be clearly reported, and percentages should be given with confidence intervals. The methods section should be detailed enough for reviewers to identify the analytical technique used and how specific variables were measured.

| Yes: Numerator, denominator, and percentages are clearly reported for dichotomous variables with enough information to calculate the confidence intervals correctly, and analyses are properly conducted. (see STROBE reporting guidelines, if necessary).  No: The numerator, denominator, or percentages are not clearly reported for dichotomous variables, authors do not provide enough information to calculate the confidence intervals correctly, or analysis are not properly conducted and likely lead to bias. | Examples for results reporting, *see STROBE guidelines if clarity of methods reporting is not clear:*   - Current (30-day) major depression prevalence was 3.8% (n = 13, 95% CI 2.2%, 6.3%), 12-month prevalence was 10.7% (n = 37, 95% CI 7.9%, 14.4%) and lifetime prevalence was 22.9% (n = 79, 95% CI 18.8%, 27.6%). The answer is: Yes. - Diagnostic interviews were conducted only for participants with a Patient Health Questionnaire (PHQ) score of at least 3. The answer is: No. |
| --- | --- |

1. **Was the response rate adequate, and if not, was the low response rate managed appropriately?**

A large number of dropouts, refusals or “not founds” amongst selected subjects may diminish a study’s validity, as can a low response rates for survey studies. The authors should clearly discuss the response rate and any reasons for non-response and compare persons in the study to those not in the study, particularly with regards to their socio-demographic characteristics. If reasons for non-response appear to be unrelated to the outcome measured and the characteristics of non-responders are comparable to those who do respond in the study (addressed in question 5, coverage bias), the researchers may be able to justify a more modest response rate.

| Yes: At least 75% of those approached with the survey provided responses and had their responses included, OR, an analysis was performed that showed no substantive differences in relevant demographic characteristics between responders and non-responders.  No: Less than 75% of those approached with the survey provided responses and had their responses included and if any analysis comparing responders and non-responders was done, it showed substantive differences in relevant demographic characteristics between responders and non-responders.  Unclear: Not enough information provided to determine. | Examples:   - The response rate was 68%; however, the researchers did an analysis and found no meaningful difference between the full study sample and the subgroup in terms of age, sex, occupation and socioeconomic status. The answer is: Yes. - The overall response rate was 65% and the researchers did NOT carry out an analysis to compare relevant demographic characteristics between the full study sample and the subgroup. The answer is: No. - The response rate was 69% and the researchers did an analysis and found meaningful differences in age, sex and socioeconomic status between the full study sample and the subgroup. The answer is: No. |
| --- | --- |

**APPENDIX 5: Adapted version of the Joanna Briggs Institute Checklist for Prevalence Studies – Factors Studies**

1. **Was the sample frame appropriate to address the target population?**

This question relies upon knowledge of the broader characteristics of the population of interest and the geographical area. The term “target population” does not mean that we would be looking for every individual with scleroderma (for example). **Instead, give consideration to specific population characteristics in the study, including age range, gender, morbidities, and other potentially influential factors.** For example, a sample frame may not be appropriate to address the target population if a certain group has been used (such as individuals with scleroderma who were aged at least 60 years) and the results then inferred to the target population (i.e., individuals with scleroderma of any age). A sample frame may be appropriate when it includes almost all the members of the target population (i.e., a census or a complete list of participants or complete registry data).

| Yes: The sampling frame was a true or close representation of the target population.  No: The sampling frame was NOT a true or close representation of the target population.  Unclear: Not enough information provided to determine. | Examples:   - The target population was the population of individuals with scleroderma of any age, sex, and disease severity, and eligible participants included all individuals with scleroderma of any age, sex, and disease severity. The answer is: Yes. - The target population was the population of individuals with scleroderma of any age, sex, and disease severity, and eligible participants included only individuals with scleroderma who were at least 60 years of age. The answer is: No. |
| --- | --- |

1. **Were study participants recruited in an appropriate way?**

Studies may report random sampling from a population, and the methods section should report how sampling was performed. Random sampling from a defined subset of the population (sample frame) should be employed in most cases, however, random probabilistic sampling is not needed when everyone in the sampling frame will be included/ analysed. For example, reporting on all the data from a good census is appropriate as a good census will identify everybody. When using cluster sampling, such as a random sample of hospitals within a region, the methods need to be clearly stated. Convenience samples, such as samples recruited from existing organizations of nurses is not considered to provide a representative sample of the base population.

| Yes: A census was undertaken, OR, some form of random selection was used to select the sample (e.g. simple random sampling, stratified random sampling, cluster sampling, systematic sampling).  No: A census was NOT undertaken, AND some form of random selection was NOT used to select the sample.  Unclear: Not enough information provided to determine. | A census collects information from every unit in the sampling frame. In a survey, only part of the sampling frame is sampled. In these instances, random selection of the sample helps minimize study bias. Examples:   - A consecutive sampling of all patients or a random sample was done. The answer is: Yes. - A convenience sample in a clinical setting was done or a Facebook advertisement was placed on scleroderma pages to encourage people to go online to respond to the survey: No. |
| --- | --- |

1. **Was the sample size adequate?**

The larger the sample, the more confidence we can have with factors that are found to be related to an outcome. Ideally, authors report conducting a sample size calculation to determine an adequate sample size. However, it is important to have enough participants and events for any variable included in the authors model. A minimum of 10 participants for continuous outcomes or 10 events (or non-events; use the smaller one) for each dichotomous outcome variable will be necessary to be deemed at “Yes” for this question.

| Yes: There are 10 participants for each variable included in the model for continuous models and 10 events per participant for models with dichotomous outcomes (or non-events; use the smaller of the two). Additionally, all categorical variables should include at least 10 participants per level.  No: There are fewer than 10 participants for each variable included in the model for continuous models and fewer than 10 events per participant for models with dichotomous outcomes (or non-events; use the smaller of the two). Or, not all categorical variables include at least 10 participants per level. | Examples:   - A model with a continuous outcome that includes 12 predictors includes at least 120 participants. And a model that includes a categorical variable, such as sex, includes at least 10 participants in both categories (i.e., n=20 male participants, n=100 female participants): Yes. - There are 12 variables included in a sample of 100 participants. Or the variable of sex, includes just 8 male participants, and 92 female participants: No. |
| --- | --- |

1. **Was there adequate coverage of potential predictors?**

Including relevant sociodemographic and disease-related factors are important when assessing findings from a study model. A minimum set of variables should be in analyses.

| Yes: Factors included in the multivariate model included at least (1) age, (2) sex, **and** (3) one disease-related variable (e.g., disease sub-type, disease severity).  No: The minimum set of socio-demographic, and disease-related variables has not been reported. | Examples:   - Age, sex, and disease-related variables, were included as predictors of increased anxiety symptoms. Yes. - Age and sex were included as predictors of anxiety symptoms, but no disease-related variables. No. |
| --- | --- |

1. **Was the response rate adequate, and if not, was the low response rate managed appropriately?**

A large number of dropouts, refusals or “not founds” amongst selected subjects may diminish a study’s validity, as can a low response rates for survey studies. The authors should clearly discuss the response rate and any reasons for non-response and compare persons in the study to those not in the study, particularly with regards to their socio-demographic characteristics.

| Yes: The response rate for the study was ≥ 75%, OR, an analysis was performed that showed no substantive differences in relevant demographic characteristics between responders and non-responders.  No: The response rate was < 75%, and if any analysis comparing responders and non-responders was done, it showed substantive difference in relevant demographic characteristics between responders and non-responders.  Unclear: Not stated. | Examples:   - The response rate was 68%; however, the researchers did an analysis and found no substantive differences between responders and non-responders in terms of age, sex, occupation, socioeconomic status, and disease severity. The answer is: Yes. - The response rate was 65% and the researchers did NOT carry out an analysis to compare relevant demographic characteristics between responders and non-responders. The answer is: No. - The response rate was 69% and the researchers did an analysis and found substantive differences in age, sex, socioeconomic status, and disease severity between responders and non-responders. The answer is: No. |
| --- | --- |

1. **Were valid methods used for the identification of the outcome variable?**

This item considers measurement or classification bias for the mental health outcome. As all studies require a validated instrument to be used, this item should be rated as **yes** for all studies.

| **Note: All studies will be YES for this question as we require a valid outcome measure.** | |
| --- | --- |
| Yes: The study instrument had been shown to have reliability and validity, e.g. test-retest, piloting, validation in a previous study, etc.  No: The study instrument had NOT been shown to have reliability or validity.  Unclear: Not stated. | Examples   - The authors used the PHQ-9, which had previously been validated. The answer is: Yes. - • The authors developed their own questionnaire and did not test this for validity or reliability. The answer is: No. |

1. **Was the mental health outcome measured in a standard, reliable way for all participants?**

This item considers how the outcome variable was collected, such as through self-report measures or for interviews, considering if the administrators had an appropriate level of education, clinical or research experience, or level of responsibility to assess an outcome.

| Yes: All self-report data were collected directly from the subjects. Any clinical interview data includes at least information about the interviewers’ level of education or training received. The same mode of data collection was used for all subjects. All aspects of this question must be present (where relevant).  No: In some instances, data were collected from a proxy (e.g., a spouse). The qualifications of clinical interviewers are not reported or not appropriate. The same mode of data collection was NOT used for all subjects. If any aspects of this item are absent, it is NO.  Unclear: Not stated. | Examples:   - All subjects completed a self-report measure from the participant themselves or were interviewed by a research coordinator with sufficient qualifications and training to administer the assessment tool that was used. The answer is: Yes. - Subjects did not all use a self-report measure, or were interviewed by an individual were their experience was not described or was not adequate (e.g., undergraduate students administered SCID depressive symptom modules). The answer is: No. |
| --- | --- |

1. **Were the variables included in the model were described as pre-specified?**

The variables included in the model are described as having been chosen a priori, ideally, with a rationale provided for the variable selected.

| Yes: Variables included in the model were stated as being selected prior to data collection, and the selection of variables was plausibly done a priori.  No: Variables included in the model were stated as being selected post-hoc, after data collection.  Unclear: Not stated. | Examples:   - Variables included in the regression model were selected a priori based on previous research highlighting the relationship between sociodemographic factors, previous mental health, and risk of health care workers in increased reports of anxiety during a health pandemic. The answer is: Yes. - Variables that were found to be significantly associated with the outcome in bivariate analyses were included in the multivariate regression model. The answer is: No. |
| --- | --- |

1. **Were appropriate data presented for all variables?**

Counts and percentages of categorical data and mean and standard deviation should be clearly reported for all variables included in the model.

| Yes: All continuous variables report (1) a measure of central tendance (e.g., mean, median) and a measure of variance (i.e., standard deviation, standard error, interquartile range, or confidence intervals) and all categorical variables, have the number of events and percentages clearly reported.  No: At least one continuous variable does not report (1) mean and a measure of variance (i.e., standard deviation, standard error, or confidence intervals) or at least one categorical variable does not clearly report the number of events and percentages. | Examples:   - Predictor variables included sex (male: 40, 40%, and female 60, 60%), PHQ-9 score (mean = 12.0, SD = 5.0), and disease type (diffuse scleroderma: 20, 20%, limited scleroderma: 80, 80%). The answer is: Yes. - Fifty percent of PHQ-9 at baseline were above the mean PHQ-9 score of the sample (13.0) as compared to 75% of scores above this mean on follow-up. The answer is: Yes. |
| --- | --- |

**APPENDIX 6: Adequacy of methods and reporting of included prevalence studies**

| **Author**  **year** | **Appropriate sample frame** | **Appropriate**  **participant**  **recruitment** | **Adequate**  **sample size** | **Participants**  **and setting**  **adequately**  **described** | **Adequate**  **response rate**  **and data**  **analysis with**  **sufficient**  **coverage** | **Valid**  **methods for**  **identification**  **of outcome**  **variable** | **Standard,**  **reliable**  **outcome**  **measurement** | **Appropriate**  **statistical**  **analysis** | **Adequate follow-up response**  **rate/ appropriate**  **management of**  **low response**  **rate** |
| --- | --- | --- | --- | --- | --- | --- | --- | --- | --- |
| Jewett/Thombs 2014/2015 | Yes | No | Yes | Yes | Unclear | Unclear | Unclear | Yes | Unclear |
| Baubet  2011  conference attendees | No | Unclear | No | No | No | No | Yes | Yes | No |
| Baubet  2011  inpatients | No | Unclear | No | No | Unclear | No | Yes | Yes | Yes |
| Jha  2022 | No | Unclear | No | No | Unclear | Unclear | Yes | Yes | Unclear |

**APPENDIX 7: Adequacy of methods and reporting of included factors studies**

| **Author**  **year** | **Appropriate sample frame** | **Appropriate**  **participant**  **recruitment** | **Adequate**  **sample size** | **Adequate coverage of potential predictors** | **Adequate response rate or adequate management of low response rate** | **Valid**  **methods for**  **identification**  **of outcome**  **variable** | **Standard,**  **reliable**  **outcome**  **measurement** | **Adequate description of variables in the model as pre-specified** | **Appropriate data presented for variables** |
| --- | --- | --- | --- | --- | --- | --- | --- | --- | --- |
| Faezi  2017 | No | Unclear | No | Yes | No | Yes | Unclear | Unclear | Yes |
| Kwakkenbos  2012 | No | Yes | Yes | Yes | Yes | Yes | Yes | Unclear | Yes |
| Thombs  2008 | Unclear | No | Yes | Yes | Unclear | Yes | Unclear | Unclear | Yes |
